# Current situation and factors influencing elderly care in community day care centers: a cross-sectional study

**DOI:** 10.3389/fmed.2023.1251978

**Published:** 2024-01-17

**Authors:** Junyu Chen, Yuan Zhu, Yulei Song, Yongqi Liang, Lulu Wang, Weitong Li, Huimei Wang, Guihua Xu

**Affiliations:** School of Nursing, Nanjing University of Chinese Medicine, Nanjing, China

**Keywords:** care, geriatric, health, nursing, elderly

## Abstract

**Background:**

The latest census data show that people over 60 years of age account for about 18.7% of the total population in China, and the aging of the population has become an irreversible trend in the 21st century. This study aimed to investigate the current status and factors influencing the care of the elderly in community day care centers in order to lay the foundation for the development of better services in community day care centers.

**Methods:**

This study was a cross-sectional survey using convenience sampling in Nanjing, China. The survey instrument was the Day care and Elderly Care Service Needs Questionnaire, which included the Ability of Daily Living Assessment (ADL), the Xiao Shuiyuan Social Support Rating Scale (SSRS) and the Day care Elderly Care Service Needs Survey Form, and a general information survey.

**Results:**

A total of 450 elderly people in day care centers were surveyed. The elderly had different levels of demand for day care services, especially regarding daily care. Correlation analyses indicated that age (*r* = 0.619), education level (*r* = 0.616), source of income (*r* = 0.582), caregiver (*r* = 0.557), satisfaction with care service (*r* = 0.603), and degree of ADL (*r* = 0.629) were correlated with the need for elderly day care services (all *p* < 0.05). The factors influencing the demand for day care services encompassed age, education level, income source, caregiver, satisfaction with service, and ADL (all *p* < 0.05).

**Conclusion:**

Elderly care services in community day care centers are mainly based on daily and spiritual comfort, and the needs of the elderly are influenced by many factors. Timely nursing care policies and measures that target these factors are needed to improve elderly care.

## Introduction

With the advent of the “silver wave,” population aging has become an irreversible trend in the 21st century (1). According to the seventh National Census, there are about 260 million people aged 60 years or older in China, accounting for 18.7 percent of the total population (2, 3). At the same time, in the era of family miniaturization, empty nesters, and economic and social transformation, the gap between social security for the elderly and the demand for old-age services has widened sharply (3–5). Still, the total amount of socialized old-age benefits is insufficient. Consequently, it is necessary to change the traditional concept of and mode of elderly care, integrate all kinds of resources from the whole community, and form a joint force to deal with the aging problem (6).

The State Council issued the Construction Plan for the Social Care Service System for Older Persons (2011–2015), which calls for the establishment of a social welfare system for the elderly “based on home care, supported by communities and supported by institutions” (7). Home-based care comprises door-to-door services, rehabilitation, and domestic services. Community-based elderly care services cover day care and home care, while institutional elderly care services include nursing homes (8). Currently, the supply of elderly care resources and services is insufficient (9). In many cities, it is difficult to find a bed in public nursing homes; the price of social nursing homes is high; and nursing resources are scarce (10). Therefore, the demand for community nursing homes has increased significantly. However, their limited service offerings create an imbalance between supply and demand (11).

With the outstanding problem of elderly care, community day care for the elderly, as a new model of elderly care, conforms to the trend of international elderly care services (12). Compared with pure elderly care institutions, it has the advantages of low fees, less investment, convenient proximity, free access, moderate space, and more complete services (13). It can maintain functional needs to a certain extent, meet psychological needs, increase social interaction, and reduce the burden on caregivers (14, 15). Therefore, the construction of community day care centers is a realistic choice to solve the problems of elderly care in our country.

The day care center is an essential support for the community elderly care system. It has been noted that in China, home care services have gradually evolved beyond life care to encompass spiritual comfort, alongside the development of community-based day service institutions (16). This study has examined the role of the community day care center from two perspectives (16). First, from the perspective of combining elderly care providers and residential places for the elderly. Second, the system of social services for the elderly is a continuum that serves the activities and care needs of older adults. One end of the continuum is connected to active assistance services for the elderly, and the other provides end-of-life services. Day care services are part of a multi-level linkage system (17).

Although the government and related departments in China promote the implementation of day care services, they have not been developed due to problems in policy implementation, such as a lack of funds, healthcare providers, and resources. The day care service system for the elderly has not been properly established. The existing literature mostly only discusses the prospect and development of day care centers, and there is little research into the essential requirements of day care services for the elderly (18–22). In addition, due to the inconsistent understanding of the demands of community day care centers and their service scope, the results of demand studies vary greatly. Several reviews have evaluated older adults’ desire for day care and its influencing factors, but the findings are different, and they highlight the need for more studies from different areas and populations (23, 24). Therefore, this study aimed to investigate the demand for daytime care services among the elderly in China, providing a reference basis for policy formulation and the further development of elderly daytime care.

## Methods

The study design was cross-sectional. In this study, all methods were performed in accordance with the relevant guidelines and regulations. The study was reviewed and approved by the Ethics Committee of Nanjing University of Chinese Medicine (approval number: KY2022374). Written informed consent was obtained from all participants.

The survey was conducted in urban areas of Nanjing, China, from 1 February to 31 March 2023. The research team conducted stratified sampling for the day care centers in this area and then randomly selected patients in each day care center. The inclusion criteria for the study population were as follows: persons aged 60 years and older who had lived in the main urban areas of Nanjing for more than six months; the elderly volunteered to participate in this study. The exclusion criteria for the study population were as follows: those with mental disease or confusion; those with a significant condition that made them unable to cooperate.

The tools used in this study were the Day care Elderly Care Service Needs Survey Form, the Ability of Daily Living Assessment (ADL) and the Xiao Shuiyuan Social Support Rating Scale (SSRS).

The questionnaire included socio-demographic information such as age, sex, family status, level of education, living conditions, sources of income, monthly income, and illness. It also asked about the elderly’s willingness to accept volunteer services, satisfaction with the content of nursing services, and reasons for dissatisfaction. Lawton and Brody developed the ADL scale in 1969 to assess the ability of older adults to live their daily lives adequately (25). The score ranges from 0 to 100. A score above 60 is considered mild dysfunction and essential self-care in daily living; a score between 60 and 41 indicates that people with moderate dysfunction need help with everyday life; a score between 40 and 21 indicates severe dysfunction, obviously dependent on everyday life; a score below 21 suggests complete disability and dependency in daily living. The higher the total score, the greater the ability to perform daily tasks. The SSRS scale is a social support rating scale developed by Xiao Shuiyuan between 1986 and 1993 to assess social support among the elderly (26). This scale has 10 items: emotional support (four items:1, 3, 4, 5), objective support (three items:2, 6, 7), and social support utilization (three items:8, 9, 10). The total score is the sum of the 10 items. The higher the score, the better the social support.

The “Day care and Elderly Care Service Needs Questionnaire” was designed to evaluate the demand for elderly care services among older adults attending community day care centers. It aims to analyze various factors, including physical, psychological, and cultural aspects, influencing their needs for care services (27). Through qualitative research, literature analysis, group discussion, and expert opinions, research tools were designed, and the steps of revision, preliminary investigation, and refinement were strictly implemented (28). In the first step, 10 experts with in-depth theoretical knowledge and practical experience in the field of elderly care were invited to evaluate the contents of the scale. The second step was to select 30 older adults as test participants, mainly to verify whether there were unclear language expressions and objectives in the questionnaire and to judge the rationality and feasibility of the project. The third step was a pre-survey. A total of 100 older adults were selected for the survey, and the reliability of the questionnaire was tested. Finally, the day care and elderly care service needs questionnaire was designed. The Cronbach’s α coefficient for the day care service demand table was 0.88, with each item ranging from 0.72 to 0.87. All values exceeded 0.70, indicating excellent reliability for this research tool and its ability to accurately reflect the day care service needs of the elderly. The fifth step was a formal investigation. 450 older adults living in day care centers, nursing homes, and homes were recruited. The standard scale included daily care (8 items), medical care (28 items), spiritual comfort (3 items), and health regimen (12 items), for a total of 51 items. All questions on the scale were closed-ended and were measured using a Likert scale (1 = Absolutely unnecessary, 2 = Not needed, 3 = neutral, 4 = needed, 5 = very needed) (29).

The research team had received formal training, served as investigators, explained the purpose and significance of the research to the participants, and distributed the questionnaires after obtaining informed consent. The participants either completed the questionnaires themselves, or the investigators read the questionnaire item by item for older adults who had difficulty filling out the forms and recorded their responses. During the interview process, observations and collections were made regarding the interviewees’ body language, home environment, and daily activities.

This study involved a questionnaire-based survey. Reports suggest that the sample size is influenced by the number of variables, ideally ranging between 5 to 10 times the number of variables. Estimating the sample size is based on the questionnaire with the highest number of items. (30, 31). In this study, there were 51 items in the questionnaire on day care and elderly care needs for the elderly. If each item is considered as an analysis variable, it is recommended to measure at least 300 samples. Taking into account a potential 20% to 30% loss in the follow-up rate, the final sample size should be adjusted to 470.

### Statistical methods

EpiData 3.1 software was used for data entry and sorting, and SPSS 23.0 was used for analysis. The socio-demographic characteristics of the subjects were determined by descriptive analysis. The total Likert scores from the Day Care and Elderly Care Service Needs Questionnaire were categorized into two groups (those who need service and those who do not need service) using the median score. The attributes in the initial data set were categorized into numerical attributes and non-numerical attributes, respectively. If the null value was numeric, the missing attribute value was filled according to the average value of the attribute; if the null value was non-numeric, the missing attribute value was filled according to the multiplicity principle in statistics with the most frequent value. Pearson or Spearman correlation analyses were performed to analyze the correlation between patient characteristics and service needs. Also, logistic regression analysis was performed to determine the significant factors associated with the need for elderly care services. All statistical tests were two-tailed, with a statistical significance of 0.05.

## Results

A total of 470 older adults were initially included in this study. After excluding 20 invalid questionnaires that were incompletely filled out, 450 valid questionnaires were obtained. The effective recovery rate of valid questionnaires was 95.74%. The general demographic and sociological aspects of 450 older adults are shown in Table 1.

**Table 1:** Status of the elderly with different sociodemographic characteristics (*N* = 450).

Variable	Characteristic	*n*	%
Sex	Male subjects	160	35.6
	Female subjects	290	64.4
Age (years)	60 ~ 70	167	37.1
	71 ~ 80	173	38.4
	81 ~ 90	108	24
	91 ~ 100	1	0.2
	>100	1	0.2
Marital status	Unmarried	3	0.7
	Married	346	76.9
	Divorced/Separated	13	2.9
	Widowed	88	19.6
Education level	Primary school and below	171	38
	Junior high school	161	35.8
	High school or technical secondary school	40	8.9
	Junior college	50	11.1
	Bachelor’s degree or above	28	6.2
Living conditions	Living alone	10	2.2
	Living with children	184	40.9
	Living with a spouse	143	31.8
	Nursing home	113	25.1
Source of income	Savings	3	0.7
	Retirement pension	132	29.3
	Pension	87	19.3
	Wages	152	33.8
	Supported by child/children	61	13.6
	State relief aid	15	3.3
Monthly income (RMB)	<1,000	117	26.0
	1,000 ~ 2000	219	48.7
	>2000	114	25.3
Walking distance (minutes)	<15	254	56.4
	15 ~ 30	145	32.2
	>30	51	11.3
Reasons for entering day care	Difficulty in receiving family care	220	48.9
	High cost of nursing homes	141	31.3
	Enjoy family fun	89	19.8
Caregiver Classification	Nurse	126	28.0
	Elderly care worker	109	24.2
	Family care	151	33.6
	Babysitting care	40	8.9
	Volunteer	24	5.3
Satisfaction with care services	Satisfied	105	23.3
	Quite satisfied	205	45.6
	Dissatisfied	140	31.1
Causes of dissatisfaction	Costly	29	20.7
	Unprofessional	23	16.4
	Limited service offerings	85	60.7
	Bad attitude	2	1.4
	Not on time	1	0.7
Willing to accept volunteer service	No	16	3.6
	Yes	434	96.4
Types of volunteer services	Care visits	141	32.5
	Phone greeting	34	7.8
	Meal delivery service	44	10.1
	Transportation and shuttle service	114	26.3
	Leisure entertainment	101	23.3
ADL (score)	Self-care (100)	187	41.6
	Mild dysfunction (>60)	210	46.7
	Moderate dysfunction (41 ~ 60)	40	8.9
	Severe dysfunction (21 ~ 40)	13	2.9

As shown in Table 2, there were statistical differences in age, education level, source of income, caregiver, satisfaction with care service, and degree of ADL between the patients who needed service and those who did not need service (all *p* < 0.05), no statistical differences were found in terms of sex, family status, living conditions, monthly income, walking distance, reasons for entering care, reasons for dissatisfaction, willingness to accept volunteer service, and types of volunteer service (all *p* > 0.05).

**Table 2:** Characteristics of patients who need care and those who do not.

Variable	Characteristic	Need care (*n* = 257)	Do not need care (*n* = 193)	t/F	*p*
Sex	Male subjects	92	68	1.446	0.109
	Female subjects	165	125		
Age (years)	60 ~ 70	81	86	2.008	0.025
	71 ~ 80	102	71		
	81 ~ 90	70	38		
	91 ~ 100	1	0		
	>100	1	0		
Marital status	Unmarried	3	0	1.387	0.116
	Married	165	181		
	Divorce/Separation	10	3		
	Widowed	79	9		
Education level	Primary school and below	114	57	1.951	0.038
	Junior high school	90	71		
	High school or technical secondary school	18	22		
	Junior college	24	26		
	Bachelor’s degree or above	11	17		
Living conditions	Living alone	6	4	2.847	0.114
	Living with children	102	82		
	Living with a spouse	63	80		
	Nursing home	86	27		
Source of income	Savings	2	1	2.797	0.018
	Retirement pension	50	82		
	Pension	41	46		
	Wages	120	32		
	Supported by child/children	30	31		
	State relief aid	14	1		
Monthly income (RMB)	<1,000	69	48	1.461	0.075
	1,000 ~ 2000	109	110		
	>2,000	79	35		
Walking distance (minutes)	<15	139	115	1.279	0.105
	15 ~ 30	82	63		
	>30	36	15		
Reasons for entering day care	Difficulty in receiving family care	109	111	1.662	0.084
	High cost of nursing homes	94	47		
	Enjoy family fun	54	35	1.745	0.014
Caregiver Classification	Nurse	66	60		
	Elderly care worker	40	69		
	Family care	123	28		
	Babysitting care	18	22		
	Volunteer	10	14		
Satisfaction with care services	Satisfied	46	59	2.866	0.107
	Quite satisfied	119	86		
	Dissatisfied	92	48		
Causes of dissatisfaction	Costly	12	17	1.185	0.312
	Unprofessional	10	13		
	Limited service offerings	55	30		
	Bad attitude	1	1		
	Not on time	1	0		
Willing to accept volunteer service	No	8	8	1.985	0.226
	Yes	249	185		
Types of volunteer Services	Care visits	87	54	1.099	0.101
	Phone greeting	10	24		
	Meal delivery service	30	14		
	Transportation and shuttle service	71	43		
	Leisure entertainment	54	47		
ADL (score)	Self-care (100)	71	116	1.839	0.002
	Mild dysfunction (>60)	135	75		
	Moderate dysfunction (41 ~ 60)	38	2		
	Severe dysfunction (21 ~ 40)	13	0		

As shown in Table 3, correlation analyses indicated that age (*r* = 0.619), education level (*r* = 0.616), source of income (*r* = 0.582), caregiver (*r* = 0.557), satisfaction with care service (*r* = 0.603), and degree of ADL (*r* = 0.629) were correlated with the need for elderly day care services (all *p* < 0.05).

**Table 3:** Correlation analysis of patient characteristics and service needs.

Characteristics	*r*	*p*
Sex	0.107	0.213
Age	0.619	0.009
Marital status	0.085	0.117
Education level	0.616	0.043
Living conditions	0.091	0.123
Source of income	0.582	0.036
Monthly income (RMB)	0.118	0.095
Walking distance	0.099	0.172
Reasons for entering care	0.104	0.078
Caregivers	0.557	0.015
Satisfaction with care services	0.603	0.047
Causes of dissatisfaction	0.126	0.068
Willing to accept volunteer service	0.201	0.113
Types of volunteer services	0.197	0.085
ADL	0.629	0.007

As indicated in Table 4, logistic regression analysis indicated that age, education level, source of income, caregiver, satisfaction with care service, and degree of ADL were the independent influencing factors on the need for elderly day care services (all *p* < 0.05).

**Table 4:** Logistic regression analysis of factors influencing the need for elderly care service.

Items	*B*	*E*	*β*	*t*	*P*	95%CI	
						Lower limit	Upper limit
Constant	56.06	4.99		12.14	<0.001	45.98	64.02
Age	−1.25	0.21	−0.32	−4.08	<0.001	−1.74	−0.11
Education level	1.02	0.26	0.23	6.49	<0.001	0.62	1.85
Source of income	−5.93	1.52	−0.23	−2.08	<0.001	−8.24	−2.91
Caregiver	4.48	1.29	0.17	2.67	0.024	0.94	7.43
Satisfaction with care service	2.19	1.06	0.22	2.14	0.018	1.04	4.27
Degree of ADL impairment	4.75	2.27	0.15	3.77	0.031	2.19	6.62

Adjusted *R*^2^ = 0.39.

## Discussion

The current study found that the elderly have different demands for day care services. According to the distribution of seniors’ needs for day care services, 21 out of 51 items have a degree of demand above the general need (>2 points). From high to low, meal services, cultural and recreational activities, emergency response services, blood glucose monitoring, blood pressure monitoring, medication management, physical rehabilitation, psychological and emotional support, health counseling, transportation services, health guidance, assistance with bathing, disease management, dietary guidance, Tai Chi and fitness exercises, dispute resolution and advocacy, health insurance, laundry assistance, diabetes management, vision and hearing assessments, environmental arrangement, oral care, and lunch services. Through the analysis of primary indicators of day care services, it appears that the elderly’s day care needs primarily revolve around daily care and emotional support, complemented by medical attention and healthcare. The number of empty-nest elderly individuals is rising. Alongside this trend, issues such as illness, financial strain, psychological pressure, depression, and loneliness are becoming increasingly prevalent (32, 33). Psychological and emotional assistance are important measures to promote the physical and mental health of the elderly. Living care and psychological support are essential to maintaining the physical and mental health of the elderly (34). With the acceleration of the aging process, many older adults sustain injuries during daily activities in addition to degeneration of organ functions (35). Therefore, the elderly urgently need professional rehabilitation, nursing, and health guidance to maintain and restore self-care abilities and promote functional rehabilitation. Preventive health care and health maintenance services also meet the needs of the elderly and become the most practical skills for disease prevention and health management of the elderly (36).

The demand for elderly day care is high (37). Older individuals with varying levels of disability exhibit distinct requirements for day care services, and those with similar disabilities often share significant demands for these services (38). It may be argued that fully independent older adults have less need for day care services, mainly spiritual comfort services, followed by daily living care and medical care. Moderately and severely dependent older adults have a tremendous need for day care services, and the range of requirements is significantly broader. Most centers focused on dietary services, blood pressure monitoring, health counseling, medication management, limb rehabilitation training, and caregiver training, but they did not include nursing techniques such as assisted sputum excretion, bedsore care, pipeline care, stoma care, enema, catheterization, bladder training, and others. This might be associated with the limited number of respondents.

The request for professional nursing services has shown a dispersed trend. For professional nursing technical services, the elderly have a wide range of relatively dispersed needs, most of which show a trend toward personalized needs and fewer overall needs (39). Currently, the development of community services is not ideal, lacking comprehensive service functionalities. Many elderly individuals choose hospitals, overlooking community medical services. This diminishes the demand for professional nursing technology in the community, limiting the development of community care services (40). However, with the further improvement of the reform of the medical system, the evolving demands of the elderly for out-of-hospital care, coupled with the enhancement of community functions, are propelling the expansion of elderly care services offered by day care centers. This expansion aims to seamlessly integrate out-of-hospital care with in-hospital services (41), fulfilling the elderly’s requirements for professional nursing and technical assistance.

The factors influencing the demand for day care services for the elderly need to be considered. Age, education level, source of living, caregiver, satisfaction with care service, and degree of ADL were the independent factors influencing the demand for day care services for the elderly. The request for spiritual comfort services among seniors decreases with age, which may be related to the deterioration of bodily organ function and the apparent decline of the elderly’s self-care ability. The education level of the elderly shows significant differences in medical care, spiritual comfort, health maintenance, and overall indicators. However, it is worth noting that the demand scores of the elderly in all indicators do not decrease with the increase in educational background. The demand scores of the group with a high school or technical secondary school education are significantly higher than those of other groups. In other words, regardless of the educational level of the elderly, the need for day care is real and must be met. When the caregivers are nurses, the elderly have more demands on each index than with other caregivers. When the caregivers are volunteers, the elderly have the lowest needs on all indicators. Therefore, the elderly are more dependent on professional caregivers (42). Compared with the requirements for daily care services, the scores for medical care services are lower, which may be related to the excellent self-care ability of the elderly.

There are some limitations to this study that must be considered. First, the needs for day care services for the elderly were assessed through a self-assessment form without expert consultation. Different older adults have different types, contents, and degrees of needs, and service contents are difficult to define (43). Experts are less aware of older people’s needs than older people themselves. Therefore, the potential sources of bias or inaccuracy must be considered. Second, many factors affect the demand for elderly care services in community day care centers (44). The factors analyzed in this study are not complete and need to be further explored. Third, evaluating the satisfaction of the elderly’s day care needs and the development model of community day care centers for the elderly is also an aspect that needs to be discussed and worked on in the future (45). Finally, the physical health of the included seniors, such as blood pressure, blood glucose, etc., was not measured in this study, and these factors may have important influences on the need for day care services for the elderly. The study results do not allow generalization as this is a local research. Therefore, larger-scale studies from different areas and populations are needed to make the results more representative and to further promote the development of long-term care services.

## Conclusion

From the point of view of the elderly, the service items from high to low are as follows: meal service, recreational activities, emergency ambulance service, blood glucose monitoring, blood pressure checks, medication guidance, health counseling, weight-bearing assistance, health coaching, bathing assistance, Tai Chi and other fitness exercises, vision and hearing evaluations, oral care, and lunch break services. Elderly day care services mainly revolve around daily care and spiritual comfort, supplemented by medical care and health care. Age, education level, source of income, caregiver, satisfaction with care services, and level of activities of daily living (ADL) are the independent factors influencing the demand for elderly day care services. The formulation of elderly care policies and caregivers should comprehensively consider those factors to provide higher-quality care services for the elderly.

## Data availability statement

The original contributions presented in the study are included in the article/supplementary material, further inquiries can be directed to the corresponding author.

## Ethics statement

The study has been reviewed and approved by the ethics committee of Nanjing University of Chinese Medicine (approval number: KY2022374). Written informed consents had been obtained from all the participants. The studies were conducted in accordance with the local legislation and institutional requirements. The participants provided their written informed consent to participate in this study.

## Author contributions

JC, YZ, HW, and GX designed the research. JC, YZ, YS, YL, LW, WL, HW, and GX conducted the research. JC, YZ, YS, and YL analyzed the data. JC, HW, and GX wrote the first draft of the manuscript. HW and GX had primary responsibility for the final content. All authors read and approved the final manuscript.
